# Verapamil Ameliorates Hepatic Metaflammation by Inhibiting Thioredoxin-Interacting Protein/NLRP3 Pathways

**DOI:** 10.3389/fendo.2018.00640

**Published:** 2018-10-31

**Authors:** Feng Zhou, Ying Zhang, Jing Chen, Yimeng Hu, Yancheng Xu

**Affiliations:** ^1^Department of Endocrinology, Zhongnan Hospital of Wuhan University, Wuhan, China; ^2^Department of Endocrinology, Puren Hospital, Wuhan University of Science and Technology, Wuhan, China; ^3^Department of Critical Care Medicine, Zhongnan Hospital, Wuhan University, Wuhan, China

**Keywords:** hepatic metaflammation, NLRP3 inflammasome, non-alcoholic fatty liver disease, verapamil, thioredoxin-interacting protein(TXNIP)

## Abstract

Activation of thioredoxin-interacting protein (TXNIP)/nod-like receptor protein 3 (NLRP3) inflammasome plays a critical role in pathogenesis of non-alcoholic fatty liver disease. This study investigated the protective effects of verapamil on hepatic metaflammation in a rodent model of high-fat (HF) diet-induced obesity (DIO). DIO was induced in a subset of mice provided with HF diet (45% kcal fat). After 10 weeks of HF diet, verapamil was administered by intraperitoneal injection. The experimental groups included the following: (1) normal diet group, (2) normal diet + treatment with verapamil (VER) group, (3) HF control group, (4) HF+VER (25 mg/kg/day) group. After 1 week of each treatment, blood and liver tissues were collected, and glucose control, serum triglyceride (TG) level, inflammation, and TXNIP/NLRP3 inflammasome were analyzed. Verapamil administration caused no alteration in food intake. HF diet impaired glucose control and increased body weight and serum TG levels. Hepatic inflammation was aggravated in HF-fed mice, as demonstrated by increased levels of pro-inflammatory markers interleukin-1β (IL-1β) and IL-18 in the liver. On the other hand, verapamil administration significantly improved glucose control, body weight, and serum TG levels. Verapamil treatment also reduced pro-inflammatory marker levels. These improvements were accompanied by alterations in activation of TXNIP/NLRP3 inflammasome. The observed results demonstrate that verapamil ameliorates hepatic metaflammation by inhibiting TXNIP/NLRP3 pathways.

## Introduction

Non-alcoholic fatty liver disease (NAFLD) and non-alcoholic steatohepatitis (NASH) are often accompanied by systemic and hepatic metaflammation (1); these conditions are some of the most common chronic liver illnesses and occur in 20% of the general population worldwide (2). Several therapeutic approaches to NAFLD have been suggested; these approaches include weight reduction (3), the use of phytonutrients (4), and pharmacological intervention (5). However, the underlying mechanisms have not been elucidated.

Inflammation is regarded a central component in NAFLD pathogenesis (6). Interleukin-1 (IL-1) cytokine family includes various inflammatory cytokines, such as IL-1β and IL-18, which are crucially involved in NAFLD pathogenesis (7, 8). Maturation and secretion of IL-1β and IL-18 mainly depend on inflammasome activation (9). Nod-like receptor protein 3 (NLRP3) inflammasome is the most extensively studied inflammasome, which comprises NLRP3, apoptosis-associated speck-like protein (ASC) and caspase-1 (Casp-1) (10). NLRP3 inflammasome activation is closely related to NAFLD development (11). Thioredoxin-interacting protein (TXNIP) is linked to NLRP3 inflammasome activation (12). Studies indicated that the TXNIP/NLRP3 inflammasome pathway is involved in NAFLD pathogenesis (13, 14). Inhibitors of TXNIP/NLRP3 inflammasome functions may therefore attenuate NAFLD.

Verapamil, a calcium channel blocker, improves hyperglycaemia and insulin resistance in metabolism syndromes (15). Shalev et al. showed that verapamil inhibits TXNIP expression in *β* cell and thus promotes *β*-cell survival and function (16). Park et al. claimed that verapamil reduces hepatic inflammation and improves metabolic homeostasis in NAFLD (17), but the related mechanism remains unknown. Therefore, we hypothesize that verapamil exerts a protective effect against high-fat (HF) diet-induced NAFLD by inhibiting TXNIP/NLRP3 pathways.

## Materials and methods

### Experimental animals

Male C57BL/6 mice (7 weeks old) were purchased from the Center for Disease Control and Prevention, Hubei, China. The mice were housed in a light- and temperature- controlled room (21–23°C; 12 h cycle) supplied with food and water as needed. HF diet feed with 45% of energy from lipids was purchased from Mediscience, Ltd. (Yangzhou, China). The Animal Care and Use Committee of the Wuhan University of Science and Technology approved all protocols (No. 02515065V). All animals received humane care according to the Principles of Laboratory Animal Care.

### Reagents

Verapamil was purchased from Shanghai Harvest Pharmaceutical Co., Ltd (Shanghai, China; No. H31021343).Antibodies against TXNIP, ASC and Casp-1 were purchased from Proteintech (Chicago, USA). NLRP3 antibody was purchased from R&D Systems (Minneapolis, MN, USA). Enzyme-linked immunosorbent assay (ELISA) kits for measurement of IL-1*β*, IL-18, and insulin contents in mice were purchased from Elabscience Biotechnology Co., Ltd (Wuhan, China). Biochemical parameter assay kits were purchased from Nanjing Jiancheng Biotechnology Co. Ltd (Nanjing, China).

### Experiment protocols

After 1 week of acclimatization, the animals were randomly divided into four groups, namely, normal diet (ND) group; normal diet + verapamil treatment (ND+VER) group; HF diet (HFD) group; and HF diet + verapamil treatment (HFD+VER) group, with ten mice in each group. After providing the mice with normal or HF diet for 10 weeks, verapamil (25 mg/kg/d) was intraperitoneally injected to the ND+VER and HFD+VER groups daily (17) for 1 week. Normal saline was administered to ND and HFD groups in the same manner. Mice were weighed weekly. At the end of the experimental period, the animals were fasted overnight and sacrificed under anesthetic conditions. Blood samples were rapidly obtained by eye removal. Livers were removed and weighed. Hepatic tissues were immediately snap-frozen in liquid nitrogen and stored at −80°C for further analysis.

### Measurement of serum content levels and cytokine assays

Blood samples were centrifuged at 4°C (2,000 r, 20 min) and measured for content levels of the following biochemical properties: glucose, triglyceride (TG), total cholesterol (TC), alanine aminotransferase (ALT), and aspartate aminotransferase (AST) by using different biochemical assay kits. IL-1β, IL-18, and insulin levels were measured by ELISA using ELISA kits. Homeostatic model assessment for insulin resistance (HOMA-IR) was calculated as follows: fasting insulin (μU/ml) × fasting glucose (mmol/l)/22.5.

### Histological analysis of liver and immunohistochemistry

Hepatic tissue samples were fixed with 10% formalin solution and embedded in paraffin. Paraffin-embedded samples were sectioned at 5 μm thickness and stained with haematoxylin–eosin (H&E). NAFLD activity score (NAS) was determined through steatosis, lobular inflammation and ballooning degeneration (18). To examine hepatic lipid deposition, frozen sections of liver tissues were stained with 0.5% oil red O reagent for 10 min and subsequently washed with isopropanol. Sections were counterstained with haematoxylin.

### Analysis of mRNA expression

Total RNA from hepatic tissues was extracted using Trizol reagent (Beyotime Biotechnology, Shanghai, China) according to manufacturer's instructions. Quality and purity of RNA were determined by U.V. spectrophotometry at 260 and 280 nm, respectively. *β*-Actin expression was used as endogenous control. Quantitative real-time polymerase chain reaction(RTPCR) was performed with an input of cDNA converted from 4 μg of total RNA. Primer sequences of mice mRNA are as follows: forward 5′-GATACCCCAGAAGCTCCTCC-3′, reverse5′-ACCTCAGTGTAAGTGGGTGG-3′ for TXNIP gene; forward 5′-CTCGCATTGGTTCTGAGCTC-3′, reverse5′-AGTAAGGCCGGAATTCACCA-3′ forNLRP3 gene; forward 5′-CTATCTGGAGTCGTATGGCTTGG-3′, reverse 5′-ATGAGTGCTTGCCTGTGCTGGTC-3′ forASC gene; forward 5′-GGCAAGCCAAATCTTTATCAC-3′, reverse 5′-GCCATCTTCTTTGTTCTGTTC-3′ for Casp-1 gene; and forward 5′-CACGATGGAGGGGCCGGACTCATC-3′, reverse5′-TAAAGACCTCTATGCCAACACAGT-3′ for *β*-actin gene. mRNA levels were evaluated by quantitative RT PCR using an ABI 7500 Fast Real-time PCR system with SYBR Green detection function(Bio, Inc.). Quantitative measurements were obtained using 2^−ΔΔ*Ct*^ method. All samples were measured in triplicate, and mean values were considered for comparative analysis.

### Western blot analyses

Liver tissues were harvested, and protein extracts were prepared according to established methods (19). The homogenates were centrifuged at 14,000 rpm for 5 min, and the supernatant nuclear extracts were then harvested and stored at −70°C. The extracted proteins were quantified by Lowry-Kalckar assays (20). Equal amounts of proteins were then separated by 10% sodium dodecyl sulfate polyacrylamide gel and then transferred to a polyvinylidene difluoride membrane. The membrane was incubated with primary antibodies at 4°C overnight and with secondary antibodies at room temperature for 2 h. Signals were detected by chemiluminescence method, and band intensities were analyzed by Quantity One Software (Bio-Rad Laboratories). Mean area density was expressed for target proteins relative to *β*-actin expression.

### Statistical analysis

Data are presented as mean ± standard deviation (SD). Measurement data were processed through Two-way analysis of variance. A Bonferroni *post-hoc* multiple comparison test was used to assess significant differences between groups. *P* < 0.05 indicates a significant difference.

## Results

### Body weights, liver weights, and food intake

At the end of the experiment, mice in the HFD group presented significantly higher average body weight than those of the ND group (^**^p < 0.01). Verapamil treatment reduced the body weight of HF-fed mice (^#^*p* < 0.01) compared with those treated with HF diet. No changes were observed in mice body weight in the ND+VER group compared with that of the ND group. Verapamil treatment showed no effect on food intake in HF diet-fed mice. Liver weights increased significantly in HFD group, compared to that of ND group (^**^p < 0.01). Verapamil treatment reduced the liver weight of HF diet-fed mice (^##^*p* < 0.01; Table 1).

**Table 1:** Effects of verapamil on body weight, food intake, and liver weight.

**Item**	**ND**	**ND+VER**	**HFD**	**HFD+VER**
Body weight	29.03 ± 1.03	29.16 ± 1.38	43.98 ± 2.31**	41.77 ± 1.73^#^
Food intake(g/day)	2.82 ± 0.22	2.89 ± 0.35	2.76 ± 0.28	2.68 ± 0.39
Liver weight	1.08 ± 0.09	1.05 ± 0.07	1.86 ± 0.13**	1.66 ± 0.11^##^

*Data are expressed as mean ± SD (n = 10 per group)*.

** *p < 0.01 vs. ND group*.

# *p < 0.05*,

## *p < 0.01 vs. HFD group*.

*ND, normal diet; VER, verapamil; HFD, high-fat diet*.

### Verapamil improves hepatic steatosis and insulin resistance in HF diet-fed mice

**Figure 1:**
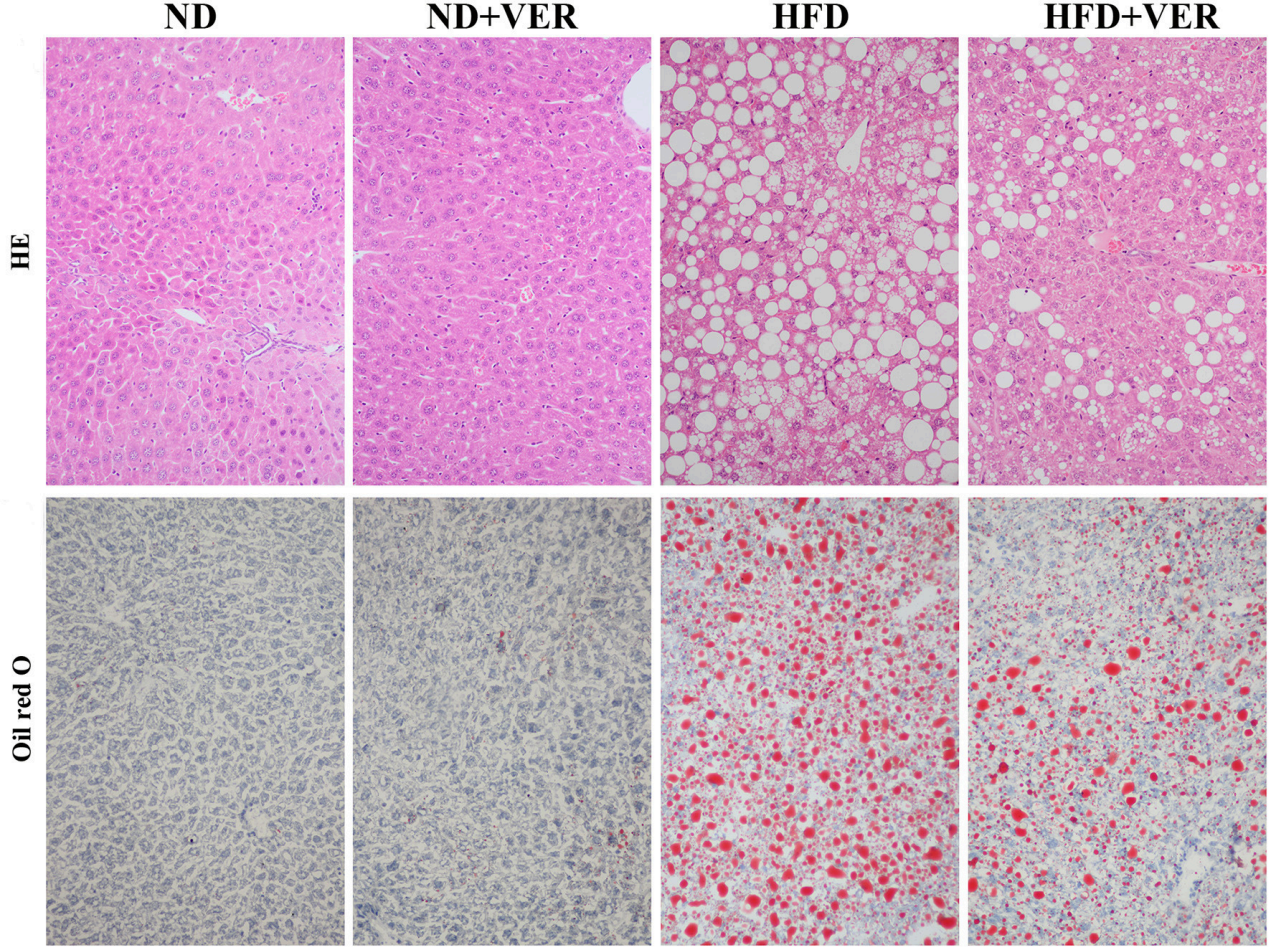
Verapamil ameliorates hepatic steatosis induced by HF diet treatment. Liver sections (*n* = 3) from each experimental group were processed for histological evaluation. Representative photographs of liver sections with H&E staining (200x) and oil red O staining (400x). ND, normal diet; VER, verapamil; HFD, high-fat diet.

High TG and TC concentrations in the serum were observed in HF-fed mice (^**^p < 0.01), but these levels decreased significantly after verapamil administration (^#^*p* < 0.01, ^##^*p* < 0.01, Table 2). Hepatic steatosis induced by HF diet was evidently ameliorated by verapamil, as indicated by normal levels of lipid accumulation and regular morphology (size and shape) of liver sections obtained from HFD+VER mice (Figure 1 and Table 3). Reduced levels of serum ALT and AST after verapamil administration supported hepatic and histological analysis results (Table 2).

**Table 2:** Effects of verapamil on serum properties of mice with NAFLD.

**Item**	**ND**	**ND+VER**	**HFD**	**HFD+VER**
TG (mmol/l)	0.32 ± 0.09	0.36 ± 0.11	0.75 ± 0.17**	0.61 ± 0.06^#^
TC (mmol/l)	2.13 ± 0.21	2.64 ± 0.17	5.44 ± 0.46**	4.34 ± 0.39^##^
Glucose (mmol/l)	5.43 ± 0.57	5.70 ± 0.37	14.52 ± 1.58**	10.94 ± 1.04^##^
Insulin (ng/ml)	0.37 ± 0.04	0.39 ± 0.05	1.31 ± 0.07**	0.76 ± 0.07^##^
HOMA-IR	2.02 ± 0.17	2.25 ± 0.29	19.03 ± 2.09**	8.43 ± 1.34^##^
ALT (U/L)	16.16 ± 3.65	17.83 ± 2.85	57.51 ± 5.65**	48.02 ± 3.74^##^
AST (U/L)	22.16 ± 4.2	24.33 ± 3.72	81.25 ± 7.04**	55.85 ± 4.29^##^

*Data are expressed as mean ± SD (n = 10 per group)*.

** *p < 0.01 vs. ND group*.

# *p < 0.05*,

## *p < 0.01 vs. HFD group*.

*ND, normal diet; VER, verapamil; HFD, high-fat diet; TG, triglyceride; TC, total cholesterol; HOMA-IR, homeostasis model assessment of insulin resistance; ALT, alanine aminotransferase; AST, aspartate aminotransferase*.

**Table 3:** Effects of verapamil on NAFLD activity score (NAS).

**Item**	**ND**	**ND+VER**	**HFD**	**HFD+VER**
Steatosis	0.00 ± 0.00	0.00 ± 0.00	2.96 ± 0.52**	1.56 ± 0.53^#^
Lobular inflammation	0.00 ± 0.00	0.00 ± 0.00	1.36 ± 0.29**	0.78 ± 0.32^#^
Hepatocellular ballooning	0.00 ± 0.00	0.00 ± 0.00	1.41 ± 0.53**	0.35 ± 0.11^##^
NAS	0.00 ± 0.00	0.00 ± 0.00	5.65 ± 0.53	3.36 ± 0.53^#^

*Data are expressed as mean ± SD (n = 10 per group)*.

** *p < 0.01 vs. ND group*.

# *p < 0.05*,

## *p < 0.01 vs. HFD group*.

*ND, normal diet; VER, verapamil; HFD, high-fat diet; NAS, NAFLD activity score*.

Levels of glucose and insulin levels and HOMA-IR index under fasting condition were measured to detect insulin resistance. The HFD group exhibited higher levels of serum glucose and insulin and HOMA-IR index than the ND group (^**^p < 0.01). On the other hand, levels of serum glucose and insulin and HOMA-IR index in the HFD+VER group significantly decreased compared with those of the HFD group(^##^*p* < 0.01).

### Verapamil inhibits activation of NLRP3 inflammasome and hepatic metaflammation in HF diet-fed mice

**Figure 2:**
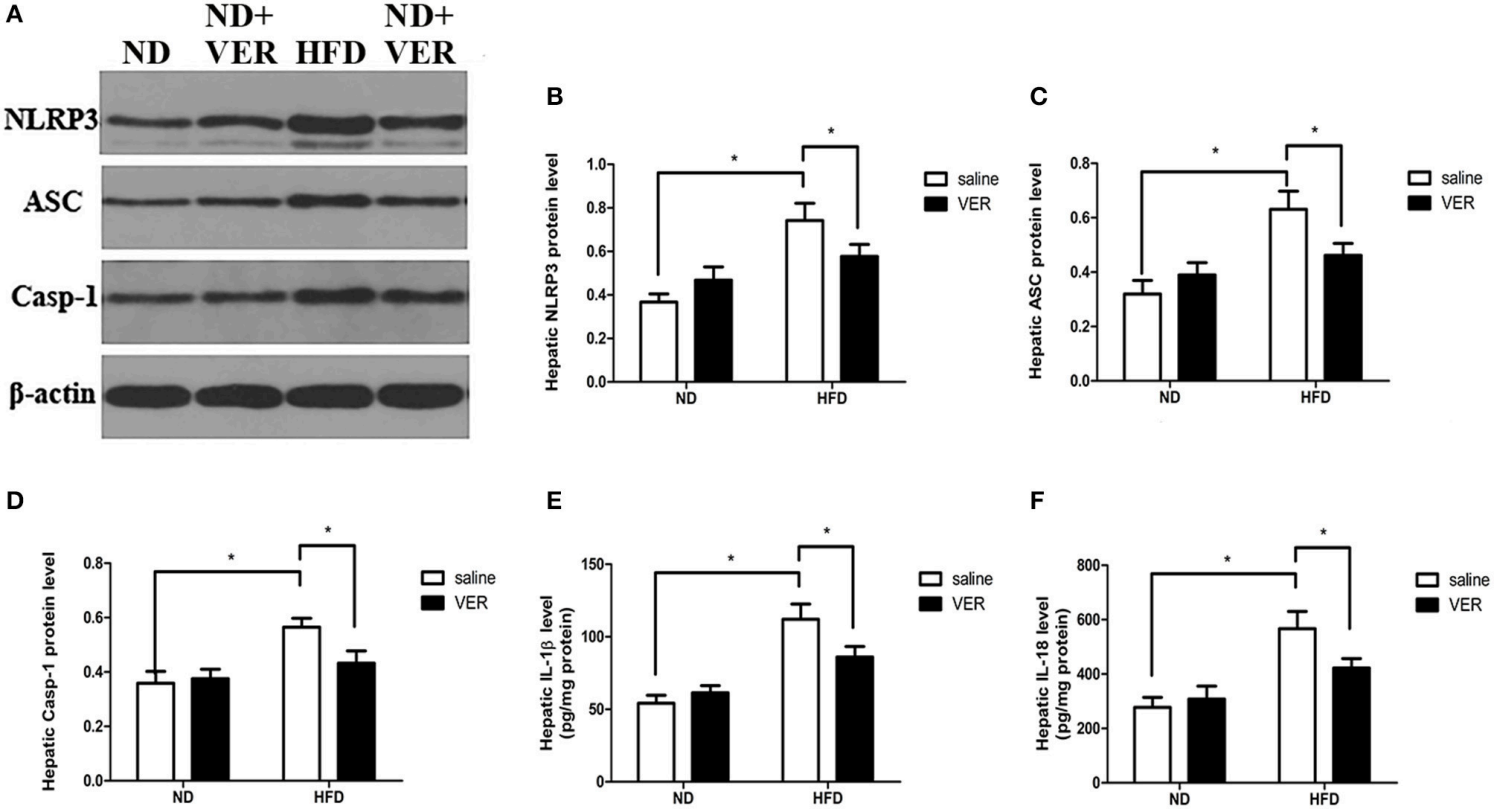
Effects of verapamil on protein expression of NOD-like receptor family, pyrin domain containing 3 (NLRP3) inflammasome complex in livers of HF diet-fed mice. **(A)** Representative Western blots for NLRP3, ASC, Casp-1 and *β*-actin. Densitometric analysis of **(B)** NLRP3, **(C)** ASC, and **(D)** Casp-1. Protein expression of each target was normalized to that of *β*-actin. Similar results were obtained among three independent experiments.The result of one of the experiments is shown. **(E)** IL-1β and **(F)** IL-18 concentrations in livers. The values were presented as means ± SD (*n* = 10 per group, *p < 0.05). ND, normal diet; VER, verapamil; HFD, high-fat diet.

Components of the NLRP3 inflammasome complex and proinflammatory markers were analyzed in livers to test whether NPRP3 inflammasome and related hepatic metaflammation participate in verapamil-mediated improvements in hepatic steatosis and insulin resistance. HF diet activated hepatic NLRP3, ASC and Casp-1 in livers of HF diet-fed mice (Figures 2A–D). Activation of NLRP3 inflammasome resulted in upregulated IL-1β levels in the HFD group (Figure 2E); these results were accompanied by high levels of pro-inflammatory cytokine IL-18 (Figure 2F). One week of verapamil administration inhibited expression of NLRP3 inflammasome components, IL-1β and IL-18, in the livers of HF diet-fed mice (Figure 2).

### Verapamil inhibits TXNIP expression in HF diet-fed mice

**Figure 3:**
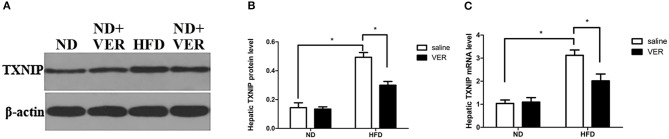
Effects of verapamil on TXNIP expression in the liver of HF diet-fed mice. **(A)** Representative Western blots for TXNIP and *β*-actin. Densitometric analysis of **(B)** TXNIP. Protein expression of the target was normalized to that of *β*-actin. Similar results were obtained among three independent experiments.The result of one of the experiments is shown. **(C)** TXNIP mRNA expression as measured by quantitative RTPCR, bars represent changes in means ± SD (*n* = 10 per group, *p < 0.05). ND, normal diet; VER, verapamil; HFD, high-fat diet.

We examined the effects of verapamil on TXNIP expression to investigate the mechanisms of NAFLD prevention by verapamil. Figure 3 shows that TXNIP expression was upregulated at protein and mRNA levels in HF-fed mice but was suppressed by verapamil treatment.

## Discussion

NAFLD is characterized by hepatic lipid accumulation and inflammation (21). In the present study, we observed the effects of verapamil on HF diet-induced NAFLD in mice. Verapamil treatment reduced serum glucose levels and improved insulin resistance, liver function, dyslipidaemia, hepatic steatosis, and inflammation. Histological analysis further revealed that verapamil significantly attenuated hepatic lipid accumulation and inflammation. However, verapamil did not have any effect on normal diet mice.

Xu et al. (22) investigated the effect of verapamil on liver fibrosis induced by multiple hepatotoxic factors in male Wistar rats with different dosages- at doses of 20, 40, and 80 mg.kg^−1^ daily for 4 weeks. It indicated that the effect was dose-dependent. A subsequent study (23) showed that verapamil was administrated as a sublethal dose (60 mg.kg^−1^) on Wistar-Albino rats for cancer therapy, but only caused miscarriage on the third day. Therefore we chose verapamil dose 25 mg.kg^−1^ as our experimental dose according to the recent research (17). We also used 25 and 10 mg.kg^−1^ in our preliminary experiment and found that the administration of 10 mg.kg^−1^ verapamil did not have protective effect on hepatic metaflammation while 25 mg.kg^−1^ verapamil could, so we finally chose the dose of 25 mg.kg^−1^.

Excessive proinflammatory cytokine-induced inflammation may play a critical role in NAFLD pathogenesis (24). Previous studies have shown that patients with NAFLD may exhibit increased IL-1β levels (25, 26). IL-1β contributed to metabolism disorder, liver injury and steatosis in animal experiments (27). Inhibition of IL-1β expression attenuates severity of hyperglycaemia in obesity (28). IL-18 levels are also associated with insulin resistance, glucolipid metabolism, and obesity (29, 30). Inhibition of IL-18 expression attenuates NAFLD development (31). In the present study, verapamil administration significantly reduced production of IL-1β and IL-18 in the HFD group. We thus recognize that protective effects of verapamil on HF diet-induced NAFLD may be ascribed to reduction of IL-1β and IL-18 levels.

The NLRP3 inflammasome pathway is a primary intracellular multiprotein inflammatory pathway of the innate immune system; this pathway responds to different exogenous and endogenous stimuli. NLRP3 inflammasome forms through activation of NLRP3 and recruitment of ASC and pro-Casp-1 (32). Such phenomena result in Casp-1 activation and subsequent processing of pro-IL-1β and pro-IL-18 to attain their active forms (33). Hepatic lipid accumulation was significantly reduced in HF diet-induced NAFLD of NLRP3^−/−^ mice (34). ASC is a crucial adaptor protein required for Casp-1 recruitment to the NLRP3 platform of inflammasome; such a process is important for activation of Casp-1and IL-1β (10). Hepatic steatosis and injury of liver function increased in Casp1(^−^/^−^) mice on HF diet compared with their wild-type counterparts (35). In the present study, verapamil treatment significantly inhibited NLRP3 inflammasome protein expression, implying that the protective effects of verapamil on HF diet-induced NAFLD may be attributed to its influence on suppression of NLRP3 inflammasome activation.

TXNIP regulates cellular oxidative stress, which has been associated with activation of NLRP3 inflammasome (36). As a result of NLRP3 inflammasome activation, TXNIP upregulation caused hepatocellular secretion of IL-1β and IL-18, thereby initiating a hepatocyte-driven sterile immune response on fructose exposure and possibly driving NASH progression (37). Xiao et al. demonstrated that inhibition of the TXNIP–NLRP3 inflammasome pathway may attenuate NAFLD progression (38). The present study demonstrated that verapamil inhibited TXNIP expression in the liver of mice with NAFLD, suggesting that the protective effects of verapamil may be due to inhibition of TXNIP/NLRP3 pathways.

Verapamil relieves hepatosteatosis of obese mice by reducing hepatic TNF- α and IL-6 levels (17), this effect may be correlated with down-regulating NF-kB activation and regulating PRMT1 and PGC-1 α (39). In our future studies, we may investigate how verapamil affects TNF- α, IL-6, NF-kB, PGC-1 α, and its underlying mechanisms.

In conclusion, verapamil treatment attenuates HF diet-induced NAFLD. The mechanisms behind the beneficial effect of verapamil are correlated with inhibition of TXNIP/NARP3 pathways, thereby remarkably reducing the expressions of proinflammatory cytokines IL-1β and IL-18. The obtained data suggest that verapamil can be a potential treatment for HF diet-induced NAFLD.

## Author contributions

FZ, YZ, JC, and YH designed the study. FZ, YZ, JC, YH, and YX conducted the experiments and wrote and revised the manuscript. YX and FZ also contributed to the design. All authors approved the final version to be published.

### Conflict of interest statement

The authors declare that the research was conducted in the absence of any commercial or financial relationships that could be construed as a potential conflict of interest. The reviewer GL and handling Editor declared their shared affiliation.
